# The Combined Effects of Co-Culture and Substrate Mechanics on 3D Tumor Spheroid Formation within Microgels Prepared via Flow-Focusing Microfluidic Fabrication

**DOI:** 10.3390/pharmaceutics10040229

**Published:** 2018-11-13

**Authors:** Dongjin Lee, Chaenyung Cha

**Affiliations:** 1 School of Materials Science and Engineering, Ulsan National Institute of Science and Technology, Ulsan 44919, Korea; dongjin625@unist.ac.kr; 2 Department of Biomedical Engineering, School of Life Sciences, Ulsan National Institute of Science and Technology, Ulsan 44919, Korea

**Keywords:** flow-focusing microfluidics, cell-laden microgel, mechanics, tumor spheroid, co-culture

## Abstract

Tumor spheroids are considered a valuable three dimensional (3D) tissue model to study various aspects of tumor physiology for biomedical applications such as tissue engineering and drug screening as well as basic scientific endeavors, as several cell types can efficiently form spheroids by themselves in both suspension and adherent cell cultures. However, it is more desirable to utilize a 3D scaffold with tunable properties to create more physiologically relevant tumor spheroids as well as optimize their formation. In this study, bioactive spherical microgels supporting 3D cell culture are fabricated by a flow-focusing microfluidic device. Uniform-sized aqueous droplets of gel precursor solution dispersed with cells generated by the microfluidic device are photocrosslinked to fabricate cell-laden microgels. Their mechanical properties are controlled by the concentration of gel-forming polymer. Using breast adenocarcinoma cells, MCF-7, the effect of mechanical properties of microgels on their proliferation and the eventual spheroid formation was explored. Furthermore, the tumor cells are co-cultured with macrophages of fibroblasts, which are known to play a prominent role in tumor physiology, within the microgels to explore their role in spheroid formation. Taken together, the results from this study provide the design strategy for creating tumor spheroids utilizing mechanically-tunable microgels as 3D cell culture platform.

## 1. Introduction

Tumor spheroids have been extensively investigated as a three-dimensional (3D) tissue model to study various aspects of cancer physiology, as they can mimic 3D solid tumor tissues more closely than conventional two-dimensional monolayers [1,2,3]. In addition, with the increasing importance of patient-specific therapeutic approach, they are also increasingly investigated as tissue-based high-throughput screening platforms for drug discovery applications that can overcome the limitations of conventional molecule-based screening. This is made possible largely due to the availability of several methods to efficiently develop spheroids in large quantities, both in templated (e.g., microwells) and suspension (e.g., hanging drop) cultures [4,5,6]. This is possible due to the highly proliferative nature and strong cell–cell interactions of various tumor cells, which are hallmarks of tumor progression, such that even under conventional in vitro cell culture conditions, the cells easily grow in numbers and often naturally aggregate to form spheroid structures.

More recently, scaffold materials such as microgels (e.g., alginate and gelatin microbeads) have been increasingly adopted to provide 3D tumor microenvironments resembling the natural extracellular matrix (ECM), in order to induce a more complex cell-matrix as well as cell–cell interactions during spheroid formation [7,8,9,10]. Due to the advancement in microfabrication technology, the size and shape of these scaffolds can be fine-tuned to generate tumor spheroids with more complex and elaborate morphologies. In addition, as the physical properties of tumor microenvironment have also been shown to influence the tumor formation and physiology, the mechanical properties of the scaffold can be controlled to influence tumor spheroid formation [11,12]. The use of scaffold also has the advantage of incorporating different types of cells in predefined numbers to generate heterogeneous spheroids [13,14]. Furthermore, the topological features of the hydrogel surface have also been shown to influence the behavior of tumor cells [15,16].

Flow-focusing microfluidics have recently gained significant interest in the field of biomedical engineering, as they can generate liquid droplets with controlled, monodisperse size and architecture with high yield and biocompatibility [17,18,19]. These droplets are used to encapsulate a wide array of biologically relevant molecules (e.g., therapeutic molecules, proteins) and species (e.g., bacterial and mammalian cells) for delivery applications. Moreover, the droplets consisting of gel-forming polymers can be crosslinked to produce microgels as cell culture platforms for tissue engineering applications [20,21,22,23,24,25].

In this study, a microfluidic device with double flow-focusing channel geometry was used to fabricate microgels encapsulated with tumor cells, in order to create uniform-sized, microgel-based 3D tumor spheroids. The aqueous droplets of methacrylic gelatin, a gel-forming polymer, dispersed with breast adenocarcinoma cells (i.e., MCF-7) were first generated by the microfluidic device, followed by photo-crosslinking to develop microgels containing tumor cells. Gelatin-based hydrogels have long been widely used as 3D cell culture platforms for tissue engineering applications. For example, gelatin, which is derived from collagen, retains cell recognition domains such as cell adhesion ligands (e.g., Arg-Gly-ASP (RGD) peptide) and matrix metalloproteinase (MMP) domains that are crucial for cellular activities such as proliferation and migration [22,23,26,27,28,29]. Due to the biocompatible and bioactive environment imparted by the crosslinked gelatin network, the cells would proliferate and eventually turn into spheroids within the microgels. There have been numerous studies on developing tumor spheroids using various cell sources, including the oft-used MCF-7 cells [30,31,32,33]. However, most of these studies were performed either in a suspension culture or on 2D substrate, and tumor spheroid formation under 3D environment has not been extensively investigated to date. This study not only allows the investigation of tumor spheroid formation within size-controlled microgels, but also explores the effect of the mechanical environment of tumor spheroid formation by controlling the mechanical properties of the microgels with the polymer concentration. Furthermore, the tumor cells were co-cultured with different types of cells—either fibroblasts or macrophages—which have been well known to be involved in tumor progression, to further influence the spheroid formation within the microgels. The results of this study are expected to delineate the combined effects of co-culture and ECM mechanics on the tumor spheroid formation.

## 2. Materials and Methods

### 2.1. Synthesis of Methacrylic Gelatin (MGel)

The methacrylate conjugation to gelatin was accomplished following a previously published method [22,23,34]. In a typical experimental set-up, gelatin (10 g, Sigma Aldrich, St. Louis, MO, USA), 4-dimethylaminopyridine (1 g, Sigma Alrdich), and 4-methoxyphenol (0.1g, Sigma Aldrich), were dissolved in 100 mL dimethylsulfoxide at 50 °C. Then, glycidyl methacrylate (4 mL, Sigma Aldrich) was slowly added and reacted for 2 days at 50 °C under dry N_2_. The product was purified by dialysis against deionized water for 2 days, changing the water three times a day, and dried by lyophilization. The chemical structure of the product was analyzed using ^1^H-NMR spectroscopy (Figure S1, Supplementary Materials).

### 2.2. Fabrication of Cell-Laden Microgels

**Figure 1:**
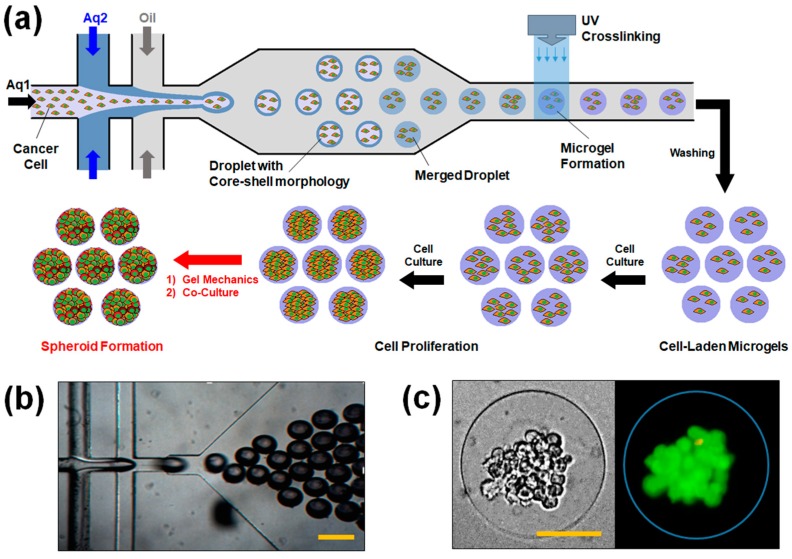
(**a**) Schematic illustration of the fabrication of cell-laden microgels using a “double” flow-focusing microfluidic device. Droplets of gel precursor solution dispersed with tumor cells are photo-crosslinked to generate the microgels. The cell-laden microgels are continuously cultured to allow the cells to proliferate and form spheroids; (**b**) A microscopic view of the microfluidic device (scale bar: 200 μm); (**c**) Representative optical (left) and fluorescent (right) images of cell-laden microgels (scale bar: 50 μm). The cells were fluorescently labeled to visualize live (green) and dead (red) cells.

The polydimethylsulfoxane (PDMS)-based microfluidic device with a “double” flow-focusing channel was fabricated using a standard photolithography and PDMS (Sylgard^®^184 Silicone Elastomer, Dow Corning, Midland, MI, USA) molding technique (detailed processing steps and channel geometry are included in Supplementary Materials) [22,23]. The channel consisted of two inlets for aqueous phases (*Aq*1 and *Aq*2) and one inlet for oil phase (*O*). *Aq*1 would become the core of a droplet, while *Aq*2 would become the shell (Figure 1a, Figure S2, Supplementary Materials). *Aq*1 and *Aq*2 consisted of MGel and 0.2% (*w*/*v*) Irgacure 2959^®^ as a photo-initiator in phosphate-buffered saline (PBS, pH 7.4). The concentrations of MGel in *Aq*1 and *Aq*2 explored in this study were 5 and 8, 7 and 10, 9 and 12, 11 and 14, and 13 and 16% (*w*/*v*), respectively. O consisted of 20% Span^®^80 (Sigma Aldrich, St. Louis, MO, USA) as a surfactant in mineral oil. In *Aq*1, human breast adenocarcinoma cells, MCF-7 (ATCC^®^, Manassas, VA, USA) were dispersed in the Aq1 at 1 × 10^7^ cells mL^−1^. For co-culture conditions, either fibroblasts (3T3, ATCC^®^) or macrophages (RAW264.7, ATCC^®^) were mixed with MCF-7 cells in varying ratios.

The fluids were injected into the microfluidic device using electronic pumps (Legato^®^100, KD Scientific, Holliston, MA, USA). The flow rate of *Aq*1 and *Aq*2 was 100 μL h^−1^, while keeping the flow rate of O at 500 μL h^−1^, resulting in droplets with an average diameter of 100 μm. The droplets were then irradiated with UV for 2 min (intensity: 200 mW, distance: 5 cm, emission filter: 250–450 nm, Model S1500, Omnicure^®^, Waltham, MA, USA) to fabricate the cell-laden microgels. The microgels were washed extensively with PBS to remove residual oil, and incubated in the cell culture medium (RPMI 1640 supplemented with 10% fetal bovine serum and 1% penicillin/streptomycin, all purchased from Thermo Fisher, Waltham, MA, USA) at 37 °C under 5% atmospheric CO_2_.

Due to the difficulty of directly measuring the mechanical properties of microgels, larger MGel hydrogel disks (8-mm diameter, 1-mm thickness) at the same MGel concentrations were fabricated by placing the precursor solution in a custom-made mold and applying the same photocrosslinking step used to develop the microgels, as stated above, and their elastic moduli were obtained from uniaxial compression experiments [35,36]. Each hydrogel disk was compressed at a rate of 1 mm min^−1^ using a universal testing machine (Model 3343, Instron, Norwood, MA, USA). The elastic modulus was calculated from the slope of a stress-strain curve at the elastic region (first 10% strain).

### 2.3. In Vitro Evaluation

#### 2.3.1. Viability and Proliferation

The viability of the cells encapsulated in microgels were evaluated using LIVE/DEAD Cell Viability Assay kit (Thermo Fisher), following the manufacturer’s instructions. Briefly, the cell-laden microgels were treated with calcein-AM and ethidium homodimer-1 to fluorescently label live (green) and dead (red) cells. The cells were visualized with a fluorescent microscope (XDS-3FL, Optika, Ponteranica, BG, Italy) and counted. The viability was reported as the percentage of live cells from the total number of cells. The viability was measured at various time points.

The proliferation rate (*k*) of encapsulated cells was determined by counting the number of live cells at various time points, and the plot of the normalized number of viable cells (*N_t_*/*N*_0_) vs. time (*t*) was fitted with the following power-law equation,

(1) $$\frac{N_{t}}{N_{0}}\;=\;2^{k\cdot t}$$

*N_t_* was the number of viable cells at time, *t*, and *N*_0_ was the initial number of viable cells at *t* = 0 [23,37].

#### 2.3.2. Immunostaining

To visualize the biomarker expression of cells encapsulated in microgels at different stages of growth, immunofluorescent labeling of CD80 and CD206 for macrophage and E-cadherin (E-cad) for MCF-7 cells was performed (detailed immunocytochemistry protocol is described in Supplementary Materials) [23,38,39]. For labeling CD80 and E-cad, hamster anti-CD80 and rat anti-E-cad were used as primary antibodies (1:250 dilution). AlexaFluor^®^568-linked anti-hamster IgG and AlexaFluor^®^488-linked anti-rat IgG were used as secondary antibodies (1:250 dilution). For labeling CD206, mouse anti-CD206 (1:250 dilution) and AlexaFluor^®^568 anti-mouse IgG (1:250 dilution) were used as primary and secondary antibodies, respectively, and labeled along with E-cad. The antibodies were purchased from Thermo Fisher. The cell nuclei were stained with 4′,6-diamidino-2-phenylindole (DAPI, Sigma Aldrich, St. Louis, MO, USA). The labeled cells within the microgels were imaged using a confocal fluorescence microscope (FV1000, Olympus, Shinjuku, Tokyo, Japan).

## 3. Results and Discussion

### 3.1. Microfluidic Fabrication of Cell-Laden Microgels

The bioactive microgels encapsulated with spheroid-forming breast tumor cells (i.e., MCF-7) were fabricated by a flow-focusing microfluidic device (Figure 1). The flow-focusing geometry of the microfluidic channel allows the formation of monodisperse aqueous droplets via shear stress applied by the oil flow. To fabricate cell-laden microgels, the aqueous droplets consisted of gel-forming macromer, methacrylic gelatin (MGel), and a photo-initiator in order to apply photo-crosslinking scheme. Herein, the “double” flow-focusing microfluidic channel geometry was utilized, in which one aqueous phase (*Aq*1) is allowed to enter the second aqueous phase (*Aq*2) before being pinched off to form droplets. Our previous studies have demonstrated that this particular channel geometry could significantly enhance the viability of the encapsulated cells by directing the cells to the center of the droplets, and eventually the microgels, by including the cells only in *Aq*1 [22,23]. This strategy minimized the cells from contacting the oil phase containing surfactants, which are well known to cause cytotoxic effects [40,41].

To help keep the cells within the core region of the droplets before employing photo-crosslinking, the MGel concentration in *Aq*2, which would become the shell region, was higher than that of *Aq*1, again following our previous studies [22,23]. With the higher viscosity of shell region, the cells within the core region would more likely remain there than move outwards, further minimizing the chances of cells contacting the surrounding oil phase. The microscopic observation of resulting microgels indeed revealed that the cells generally remained within the core region, even after the core and shell regions eventually merged via diffusion and photo-crosslinked to form microgel (Figure 1c).

One of the advantages of flow-focusing microfluidics in creating droplets is the precise control of size by simply adjusting the flow rates of aqueous and oil phases. Also, the droplets are monodisperse in size under the given flow rates, as shown in Figure 1b, especially compared with those created by non-specific high shear (e.g., sonication) which results in wider size distributions [42]. Here, the ratio of aqueous to oil flows was kept at 0.2, which resulted in droplets with an average diameter of 100 μm.

### 3.2. Effect of Microgel Mechanics on Tumor Spheroid Formation

**Figure 2:**
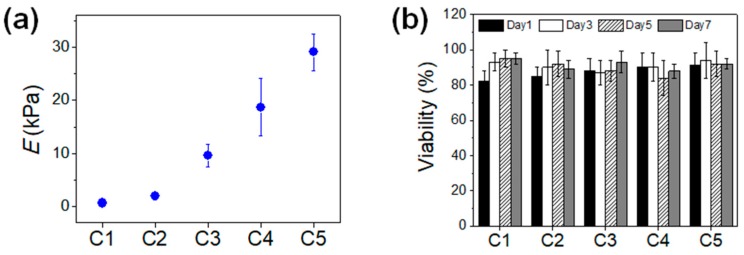
(**a**) Elastic moduli (*E*) of MGel hydrogels at varying concentrations; (**b**) The viability of MCF-7 cells encapsulated in microgels at varying MGel concentrations, measured at various times up to 7 days.

To investigate the effect of microgel mechanics on the viability and proliferation of the encapsulated cells, the mechanical properties of the microgels were controlled by varying the MGel concentrations. Five different sets of MGel concentrations for *Aq*1 and *Aq*2 were explored: 5% and 8% (C1), 7% and 10% (C2), 9% and 12% (C3), 11% and 14% (C4), and 13% and 16% (C5). The overall MGel concentration after the merging of core and shell regions were estimated to be 6.2% (C1), 8.2% (C2), 10.2% (C3), 12.2% (C4), and 14.2% (C5), determined based on the relative amounts of core and shell regions (detailed calculations are provided in Supplementary Materials). Due to the difficulty of directly measuring the mechanical properties of microgels, larger hydrogels at the same MGel concentrations via photo-crosslinking were separately fabricated, and their elastic moduli were obtained from uniaxial compression (Figure 2a). The moduli could be controlled in a wide range, from 0.7 kPa to 30 kPa, by varying the MGel concentration.

**Figure 3:**
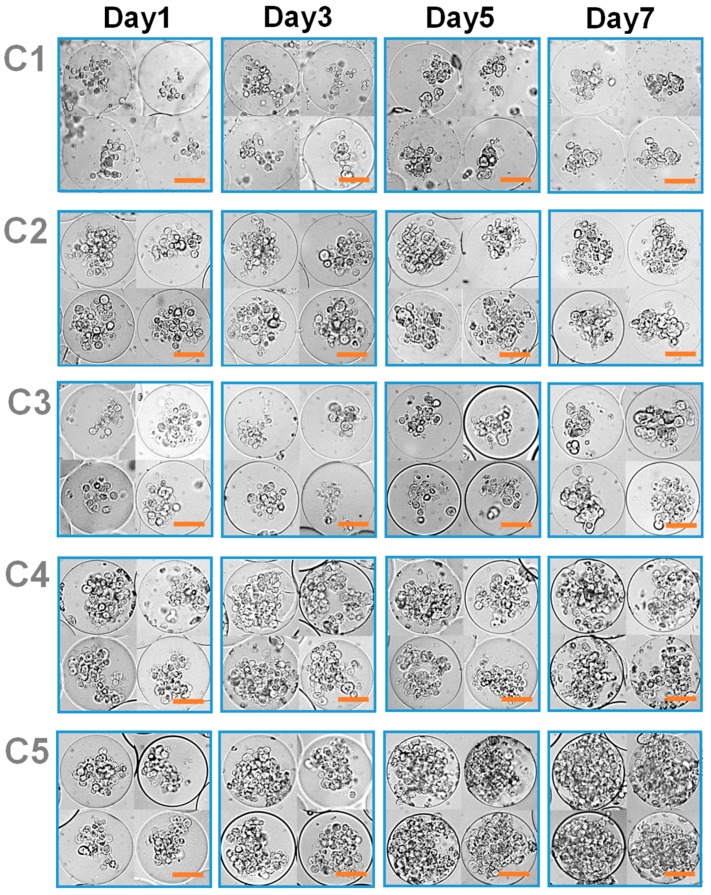
Microscopic images of cell-laden microgels at various mechanical stiffness (from C1 to C5), controlled by MGel concentration, cultured over time (scale: 50 μm).

**Figure 4:**
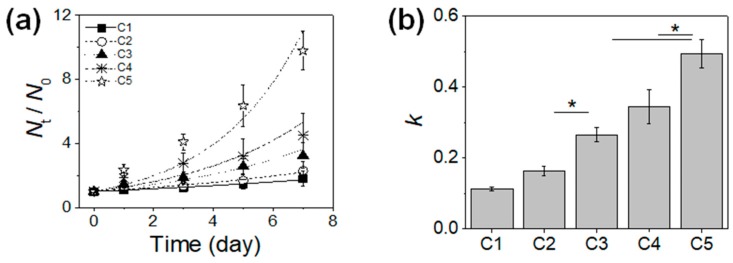
(**a**) The number of live MCF-7 cells at various times (*N_t_*) normalized with the initial number of live cells (*N*_0_) plotted over time; (**b**) The proliferation rate (*k*) obtained by fitting the plot in (**a**) with Equation (1). (* *p* < 0.05, *n* = 10).

The MCF-7 cells encapsulated in the microgels with varying mechanical stiffness were cultured, and their viability and proliferation were measured. The cell viability was well maintained regardless of the microgel conditions, all above 80%, demonstrating the biocompatibility of the microfluidic process and the 3D microenvironment provided by the microgels (Figure 1c and Figure 2b). However, there was a significant difference in cell proliferation in response to varying mechanical stiffness of the microgels. The cell proliferation increased substantially with increasing mechanical stiffness, as identified by the microscopic observation (Figure 3). To further quantify the rate of proliferation at different microgels, the number of cells were counted at various times throughout the cell culture, and the plot of number of cells vs. time was fitted with a population doubling power-law model to obtain the proliferation rate (*k*) (Figure 4). In accordance with the microscopic images, the *k* values increased with mechanical stiffness of the microgels. Previous studies have similarly demonstrated that the proliferation of MCF-7 cells was enhanced on stiffer substrates [43]. However, the majority of the studies have been performed on the surface (2D). Since medium diffusion into the microgel and the available inner space in the microgel for cell growth becomes more limited at higher stiffness, which would be viewed as deterrent for cellular growth, the increase in proliferation under those circumstances revealed that the mechanotransduction imparted by the higher microgel stiffness had a significant influence on proliferation. Moreover, the overall microgel dimension did not change during the proliferation, suggesting that the cells could remodel the internal structure to accommodate the increasing number of cells.

**Figure 5:**
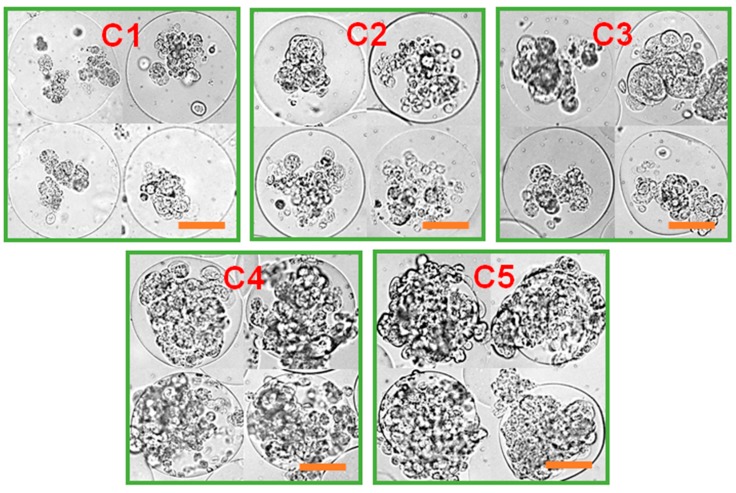
The tumor spheroid formation within microgels with varying mechanics after 14 days of cell culture (scale: 50 μm). A collection of smaller spheroids was developed within the microgel.

With the continued cell culture up to 2 weeks, the cells formed a collection of smaller spheroids within the microgels, in which the cell clusters organized into more well-defined spherical entities (Figure 5). The size of these spheroids was larger at higher microgel stiffness, suggesting that greater number of cells during the proliferation naturally led to the formation of larger spheroids. Especially at C4 and C5, the cells outgrew the size of the microgels, such that some of the cells could migrate out of the microgels. Overall, these results highlighted that the MCF-7 cells within the microgels showed higher proliferation at greater microgel stiffness, and the cells eventually turned into spheroids. However, it should be noted that the continued cell proliferation did not lead to a singular large spheroid in a microgel, rather a number of smaller spheroids co-existing within the microgel.

### 3.3. Effect of Co-Culture on Tumor Spheroid Formation

**Figure 6:**
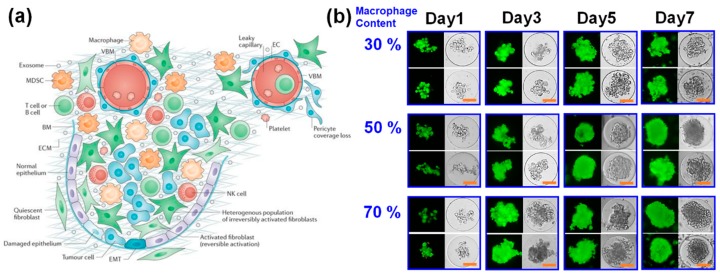
(**a**) Schematic illustration of tumor microenvironment, consisting of multiple types of cells. Reprinted with permission from ref. [46]. Copyright 2016 Springer Nature; (**b**) Optical (left) and fluorescent (right) microscopic images of microgels encapsulated with varying amounts of macrophages co-cultured with MCF-7 cells (scale bar: 50 um). The cells were fluorescently labeled to visualize live (green) and dead (red) cells.

It has been widely reported that the many tumor tissues contain a heterogeneous mixture of different cell types which help promote tumor growth and metastasis (Figure 6a) [44,45,46]. For example, tumor-associated macrophages are found in most solid tumors, and involved with angiogenesis, immune suppression and tissue remodeling which help tumor progression [44,45]. Fibroblasts are also similarly recruited by tumor cells and become activated as tumor-associated fibroblasts which similarly aid in tumor growth by promoting angiogenesis and tissue remodeling [46,47]. Therefore, it was hypothesized that the presence of these supporting cells would help turn the MCF-7 cells into a larger and more mature (compact) tumor spheroid within a microgel. To investigate the role of supporting cells on the tumor spheroid formation within microgels, either macrophages or fibroblasts were co-cultured with MCF-7 cells within the microgels, and tumor spheroid formation was monitored.

First, the MCF-7 cells were co-cultured with macrophages (RAW264.7 cell line) in microgels at different macrophage contents (30%, 50%, and 70% macrophages) (Figure 6b). The MGel concentration of the microgel was fixed at C3. Regardless of the cell compositions, the viability of the cells was high and well maintained throughout the culture. In addition, the cells all underwent significant proliferation over the course of cell culture. At lower macrophage content (30%), the cell proliferation and spheroid morphology were similar to those with only MCF-7 cell, in which several smaller tumor spheroids were formed within a microgel. When the amount of macrophages was increased (50% and 70%), the cells proliferated at faster pace and transformed into a large, single tumor spheroid with greater uniformity within a microgel. Remarkably, at the highest macrophage content (70%), the tumor spheroids were more readily formed by only day 3 of culture, further highlighting the role of macrophages in promoting tumor spheroid formation. In all conditions, there were filopodial projections the periphery of tumor spheroids, a hallmark of tumor invasion and metastatic potential [48,49,50]. This observation further gave evidence that the macrophages were actively involved with the tumor progression and spheroid formation.

Next, MCF-7 cells were co-cultured with fibroblasts in the microgels at different ratios and the tumor spheroid formation was examined (Figure S3, Supplementary Materials). At lower fibroblast content (30%), the cell proliferation was not significantly different from that with only MCF-7 cells. In addition, the spheroids formed within a microgel were smaller than those co-cultured with macrophages. At higher fibroblast content (50%), more well-defined and larger tumor spheroids were formed. However, the tumor spheroids were markedly smaller, and the proliferation was slower than those co-cultured with macrophages. Moreover, at the highest fibroblast content (70%), the cell proliferation and tumor spheroid formation was reduced compared with those with lower fibroblast content. This result suggested that the effect of fibroblast on promoting tumor spheroid formation may not have been as potent as the macrophages, and the initial reduced number of MCF-7 cells in the microgels with higher fibroblast content likely led to slower tumor progression. Taken together, the tumor spheroid formation was greatly aided by the presence of supporting cells, and this enhancement effect was varied depending on the cell type and density.

### 3.4. Combined Effect of Co-Culture and Microgel Mechanics on Tumor Spheroid Formation

The results presented above clearly demonstrated that both the mechanical properties of the surrounding matrix and the presence of supporting cells play important roles in tumor spheroid formation within the microgels. Therefore, to elucidate their combined effects, the co-culture of MCF-7 cells with supporting cells within microgels having varying mechanical properties was performed, and the change in tumor spheroid formation was evaluated.

**Figure 7:**
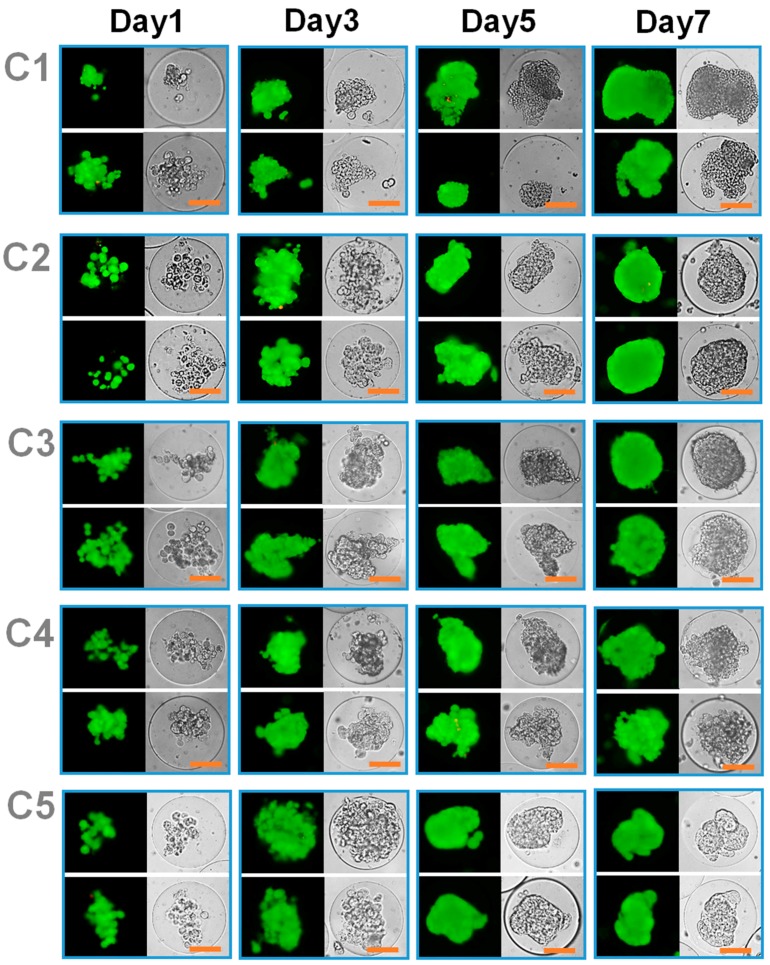
Optical (**left**) and fluorescent (**right**) microscopic images of MCF-7 cells and macrophages (5:5 ratio) co-encapsulated in the microgels with various mechanical stiffness (from C1 to C5) (scale bar: 50 um). The cells were fluorescently labeled to visualize live (green) and dead (red) cells.

The MCF-7 cells alone in microgels with varying mechanical stiffness demonstrated increase in proliferation with increasing mechanical stiffness, as demonstrated in Figure 3 and Figure 4. With the co-culture with macrophages, the trend in proliferation rate was similar, in which the cell proliferation increased with the mechanical stiffness of the microgels, but the cell proliferation was greatly enhanced at lower mechanical stiffness (C1 and C2) (Figure 7). In C1, the cells continued to proliferate and eventually broke out of the microgel, due to the structural weakness. However, the cells were closer to aggregates that continued to spread without forming a compact, well-defined spherical form that defines tumor spheroids. However, beginning with C2, the larger tumor spheroid that covers the entire microgel volume with the peripheral filopodial projection was shown. With increasing mechanical stiffness of the microgels, the tumor spheroids formed more quickly, such that larger tumor spheroids began to form only after day 3 of culture at C4 and C5. However, at the highest mechanical stiffness (C5), even though the tumor spheroid formation occurred earlier than other conditions, the size of spheroids were smaller, likely due to the increased mechanical strength of the microgels likely increased the metabolic stress and also acted as a physical barrier against forming larger spheroids [11]. Regardless, at a wide range of mechanical stiffness of the microgels, the cells all proliferated and formed a large, compact spheroid within the microgels rather than several smaller spheroids, further establishing the important role of co-cultured macrophage on the tumor spheroid formation.

**Figure 8:**
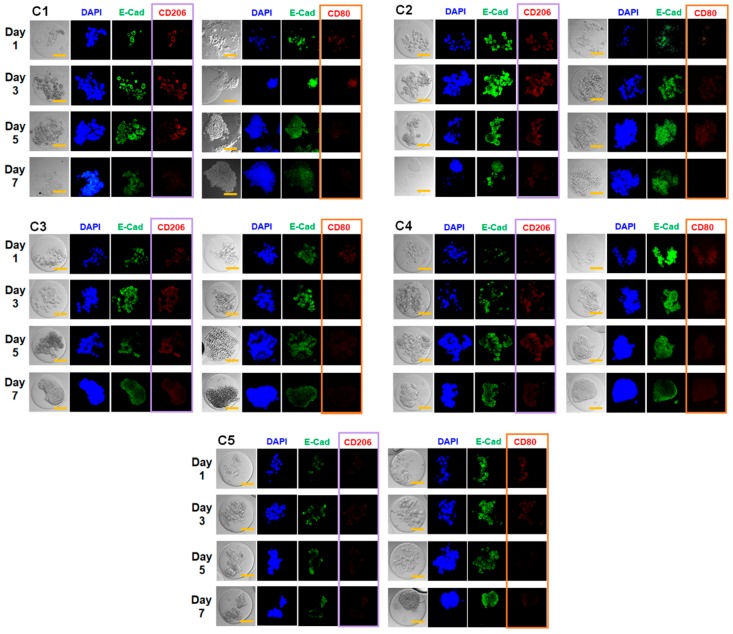
Immunocytochemical analysis of MCF-7 cells and macrophages encapsulated in microgels with varying mechanical properties (from C1 to C5). At various times up to 7 days, E-cadherin (E-cad), CD206, and CD80 were fluorescently labeled (scale bar: 50 μm). 4′,6-diamidino-2-phenylindole (DAPI) was used to label cell nuclei.

Immunocytochemical analysis of the characteristic biomarkers of MCF-7 cells and macrophages within the microgel was performed to further analyze their biochemical changes during the tumor spheroid formation (Figure 8). For MCF-7 cells, the expression of E-cadherin (E-cad), a calcium-dependent transmembrane protein responsible for cell–cell junction and communication and known biomarker for epithelial cells, was targeted and analyzed, since the expression behavior of E-cad in breast mammary cells is well known to be altered and often down-regulated, which leads to tumor progression via epithelial-to-mesenchymal transition [51,52]. For macrophages, CD206 (an M2 phenotype marker) and CD80 (an M1 phenotype marker) were targeted to monitor the degree of macrophage activation (‘Mϕ polarization’) during tumor spheroid formation. For tumor-associated macrophages, their M1 phenotype, which is involved with inflammatory and tumor-suppressive activities, is often down-regulated, and their M2 phenotype, with anti-inflammatory and immune-suppressive potential, is up-regulated, helping tumor progression [53]. Regardless of the microgel stiffness, the E-cad expression per cell significantly decreased over time, which was in line with many previous studies demonstrating their down-regulation. This suggested that the MCF-7 cells within the microgels became highly tumorigenic during their proliferation and spheroid formation. In addition, the CD206 expression was generally much larger than that of CD80 from C1 to C3, which corroborate with previous reports showing tumor-associated macrophages acquire M2 phenotype to promote tumor progression. The concurrent decrease in CD80 expression over time and the generally diminished level of CD80 expression regardless of gel mechanics were also indicative of the M1 suppression, which coincided with the increased CD206 expression and further highlighted the tumor-promoting role of macrophages within tumor microenvironment.

Interestingly, the relative expressions of CD206 and CD80 were dependent on the gel mechanics. For example, CD206 expression was much lower at higher gel stiffness (C4 and C5) as compared to lower stiffness (from C1 to C3). Also, the initial CD80 expression at day 1 was higher than CD206 expression at C4 and C5, although it decreased significantly afterwards and CD206 expression increased over time. This indicated that at higher gel stiffness, the macrophage favored M1 phenotype, but the presence of tumor cells influenced the Mϕ polarization more towards tumor-promoting M2 phenotype. In addition, while the CD206 expression showed increase over time at C4, it remained substantially low throughout the period at C5, possibly due to the limited permeability of microgels at higher stiffness suppressing Mϕ polarization itself. These results highlight the gel mechanics as well as the presence of tumor cells combine to influence the macrophage activities. Taken together, these results evidently established the synergistic role of co-culture with macrophages and tunable mechanical properties of microgels on improving the tumor spheroid formation in microgels.

## 4. Conclusions

There is a growing interest in utilizing tumor spheroids as 3D tissue structures as high-throughput screening platforms for cancer therapeutic development, because tissue-based platforms provide more in-depth biological information than conventional target molecule-based platforms. Also, the tumor spheroids can be efficiently produced in large quantities using various types of cells including those directly obtained from patients. Although several methods are available to generate spheroids, it is still a challenge to create them within a more physiologically relevant tissue microenvironment (i.e., ECM) with tunable physical properties that can more closely mimic different stages of cancer. Therefore, in this study, tumor spheroids were developed within size-controlled microgels with tunable mechanical properties as a 3D cell culture platform via a flow-focusing microfluidic fabrication. The double flow-focusing channel geometry allowed biocompatible encapsulation of cells inside aqueous droplets of gel-forming precursor solution, which were then photo-crosslinked to form cell-laden microgels. With breast adenocarcinoma cells (MCF-7), the cells encapsulated inside the microgels showed high viability throughout the cell culture regardless of the mechanical properties of microgel. However, the rate of proliferation was highly dependent on their mechanical properties; the cells proliferated faster within microgels with higher mechanical stiffness. MCF-7 cells alone did not lead to a mature spheroid within a microgel, in which all the cells form a large, compact, and well-defined spherical cell cluster, but rather a collection of smaller cell aggregates were formed regardless of the microgel stiffness. However, when MCF-7 cells were co-cultured with supporting cells (macrophages or fibroblasts, well known to be involved with tumor progression), the cells within a microgel proliferated and turned into a mature spheroid regardless of the microgel stiffness, though their rate and extent of spheroid formation was dependent on the microgel stiffness. Taken together, the microfluidic fabrication of cell-laden microgels with varying mechanical properties coupled with providing supporting cells to control tumor spheroid formation is expected to be an efficient strategy of generating a wide array of heterogeneous 3D tumor spheroids as platform for drug screening applications as well as fundamental biological investigation.

## Supplementary Materials

The following are available online at http://www.mdpi.com/1999-4923/10/4/229/s1. Table S1. The concentrations of core (C_c_) and shell (C_s_) regions of droplets, and the final concentration of droplets after merging (C_T_). Figure S1: ^1^H-NMR spectrum of methacrylic gelatin (MGel). Figure S2: Schematic illustration of double flow-focusing channel geometry of the microfluidic device used to generate cell-laden microgels. Figure S3: Optical and fluorescent microscopic images of microgels encapsulated with varying amounts of fibroblasts co-cultured with MCF-7 cells.
