# Supplementary material for: The Combined Effects of Co-Culture and Substrate Mechanics on 3D Tumor Spheroid Formation within Microgels Prepared via Flow-Focusing Microfluidic Fabrication
Source: Pharmaceutics. 2018 Nov 13;10(4):229. doi: 10.3390/pharmaceutics10040229 (PMC6321249; doi:10.3390/pharmaceutics10040229)
Supplement: Supplementary file 1 [file pharmaceutics-10-00229-s001.pdf]

pharmaceutics

Supplementary Materials: The Combined Effects of Co-Culture and Substrate Mechanics on 3D Tumor Spheroid Formation within Microgels Prepared via Flow-Focusing Microfluidic Fabrication

Dongjin Lee [1] and Chaenyung Cha [1,2,*]

[1] School of Materials Science and Engineering, Ulsan National Institute of Science and Technology, Ulsan 44919, Korea; dongjin625@unist.ac.kr
[2] Department of Biomedical Engineering, School of Life Sciences, Ulsan National Institute of Science and Technology, Ulsan 44919, Korea
* Correspondence: ccha@unist.ac.kr; Tel.: +82-52-217-5328

1. Materials and Methods

1.1. Fabrication of a Microfluidic Device

The silicon master for the PDMS-based microfluidic device was fabricated on a silicon wafer using a standard photolithography. Briefly, SU-8 100 (MicroChem Corp.) as a photoresist was first spin-coated on a silicon wafer at 1750 rpm for 30 seconds, and then baked at 65 °C for 20 minutes, followed by 95 °C for 50 minutes to harden the photoresist. The height of the photoresist became approximately 150 µm. The photomask with a pattern for the microfluidic channel was placed on top of the wafer, and irradiated with UV to fabricate the patterned area. After baking at 95 °C to further strengthen the patterned area, the wafer was placed in SU-8 developer to remove the non-fabricated area. The wafer was washed with isopropanol and dried. The channel schematic is shown in Figure S2.

PDMS elastomer was fabricated on top of the silicon master (base:curing agent = 10:1, Sylgard®184 Silicone Elastomer Kit, Midland, MI, USA). The PDMS mixture was first degassed under vacuum, and cured for 3 hours at 80 °C. The PDMS elastomer with the channel pattern engraved on the surface was detached from the master, and fluid inlets and outlets were created using a hole puncher (0.5 mm diameter). Finally, a glass slide and the PDMS were treated with oxygen plasma (Harrick Plasma) for 30 seconds and permanently bonded to each other to fabricate the PDMS microfluidic device. To inject fluids into and collect the fluid from the microfluidic device, plastic tubings (Tygon®, Saint-Gobain Performance Plastics, Merrimack, NH, USA) were connected to the inlets and outlet of the microfluidic device.

1.2. Immunocytochemistry

Immunocytochemical labeling of cells encapsulated in microgels was performed to visualize the characteristic biomarkers. Briefly, a cell-laden microgel sample was first fixed in 4 % formaldehyde solution overnight at 4 °C. After washing with PBS, the sample was incubated in a PBS-DS (5 % donkey serum in PBS) as a blocking solution for 30 minutes. After removing the blocking solution, the sample was incubated with primary antibodies for 2 hours at room temperature. After removing the antibody solution and washing with PBS, the sample was postfixed with 4 % formaldehyde for 5 minutes. After washing with PBS, the sample was treated with 0.3% Triton X-100/PBS-DS (PBST-DS) for 30 min. After washing with PBS-DS, the sample was incubated with fluorophore-conjugated secondary antibodies and 4′,6-diamidino-2-phenylindole (DAPI, 100 ng mL^{-1}) for 2 hours at room temperature. After removing the antibody solution and washing with PBST 3 times, the fluorescent image of the hydrogel surface was captured with a confocal microscope (FV1000, Olympus, Shinjuku, Tokyo, Japan).

1.3. Determination of Droplet Concentrations

The overall MGel concentration of the droplet after the merging of core and shell regions was estimated by the volumes of core and shell regions determined from the microscopic observation. Briefly, there was a clear border between core and shell regions, which are *Aq*1 and *Aq*2 phases, respectively, immediately after droplet formation. The volumes of the core (V_c) and the shell (V_s) was calculated, and their relative portions were used to calculate the overall concentration (C_T),

$$V_C = \frac{4}{3}\pi r_C^3, \quad V_S = V_T - V_C, \quad C_T = \frac{V_C}{V_T}C_C + \frac{V_S}{V_T}C_S \ ,$$

V_T was the total droplet volume, C_c and C_s were the concentrations of core and shell, respectively, and r_c was the radius of the core. In this study, the average V_c/V and V_s/V were 0.6 and 0.4. The C_T are calculated as follows:

Table S1. The concentrations of core (C_c) and shell (C_s) regions of droplets, and the final concentration of droplets after merging (C_T).

Conditions	C_c (%)	C_s (%)	C_T (%)
C1	5	8	6.2
C2	7	10	8.2
C3	9	12	10.2
C4	11	14	12.2
C5	13	16	14.2

2. Supplementary Figures

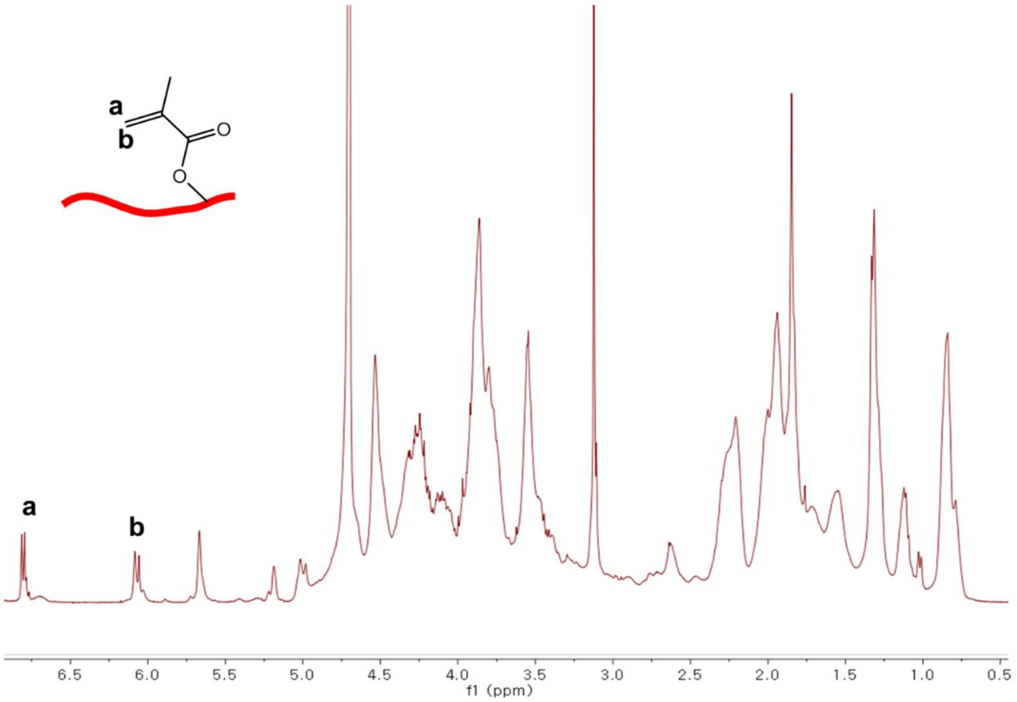

Figure S1. ^{1}H-NMR spectrum of methacrylic gelatin (MGel). Characteristic peaks (**a** and **b**) of methacrylate are noted.

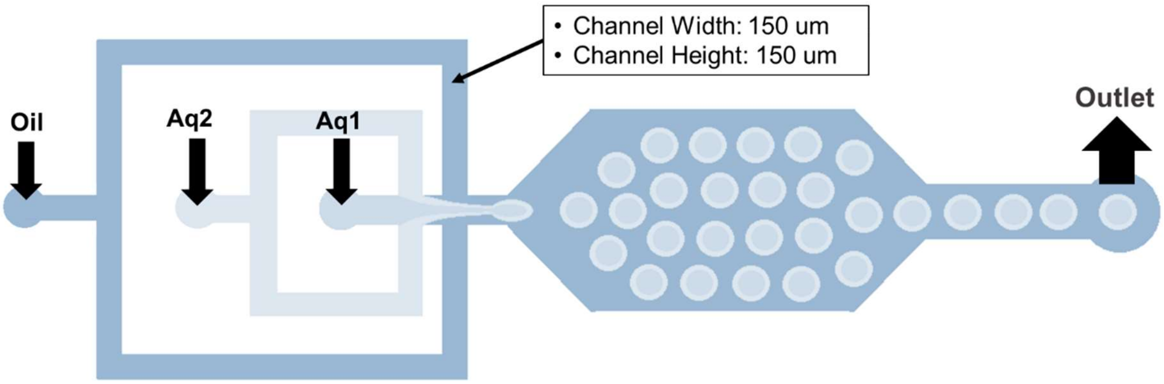

Figure S2. Schematic illustration of double flow-focusing channel geometry of the microfluidic device used to generate cell-laden microgels.

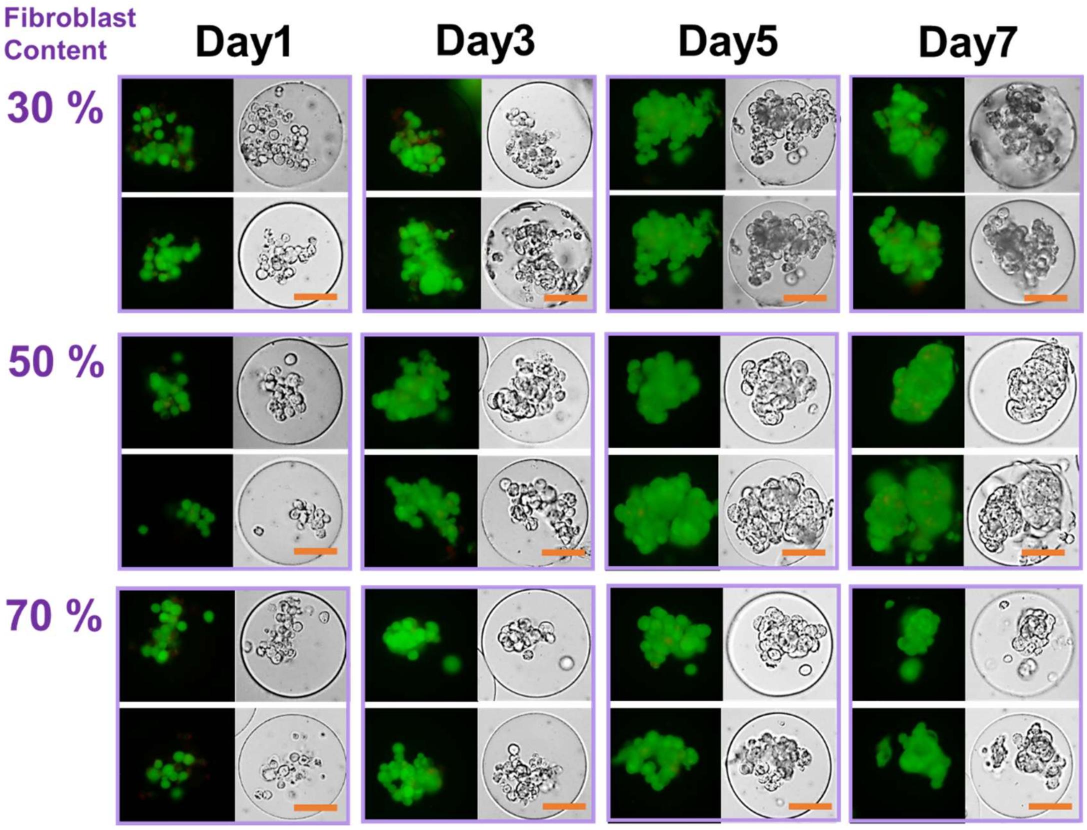

Figure S3. Optical (**left**) and fluorescent (**right**) microscopic images of microgels encapsulated with varying amounts of fibroblasts co-cultured with MCF-7 cells (scale bar: 50 μm). The cells were fluorescently labeled to visualize live (green) and dead (red) cells.
